# Recurrent pneumothoraces caused by a high-grade lung carcinoma with trophoblastic differentiation: a case report

**DOI:** 10.3389/fonc.2024.1462865

**Published:** 2024-12-24

**Authors:** Márton Csaba, Zsolt Megyesfalvi, László Báthory-Fülöp, Tamás Pintér, László Agócs, Balázs Döme, Ferenc Rényi-Vámos, Áron Kristóf Ghimessy

**Affiliations:** ^1^ Department of Thoracic Surgery, National Institute of Oncology (NIO), Budapest, Hungary; ^2^ Thoracic Surgery Clinic, Semmelweis University, Budapest, Hungary; ^3^ Department of Thoracic Surgery, National Koranyi Institute of TB and Pulmonology, Budapest, Hungary; ^4^ Department of Pathology, National Institute of Oncology (NIO), Budapest, Hungary; ^5^ Department of Oncology, National Institute of Oncology (NIO), Budapest, Hungary; ^6^ Translational Thoracic Oncology Laboratory, Clinical Department of Thoracic Surgery, University Clinic for General Surgery, Medical University of Vienna, Vienna, Austria; ^7^ National Tumor Biology Laboratory, National Institute for Oncology (NIO), Budapest, Hungary

**Keywords:** non-gestational trophoblastic tumor, pneumothorax, VATS, wedge resection, chest pain, case report

## Abstract

Gestational trophoblastic neoplasms are tumors that occur during pregnancy, while non-gestational trophoblastic tumors have a similar histology but are present outside of gestation. Literature reports several cases of non-gestational trophoblastic tumors of primary pulmonary origin, which pose diagnostic challenges and are associated with a poor prognosis. This report details a case of somatic high-grade carcinoma with trophoblastic differentiation primarily manifesting in the left lung with recurrent pneumothoraces. The tumor was initially diagnosed as a poorly differentiated pleomorphic carcinoma and was treated with paclitaxel and pembrolizumab, followed by the EMA-CO/EP regimen after the detection of liver, lung, and brain metastases. Despite initial treatment responses, the disease progressed with widespread metastases and severe complications, including myelotoxicity, empyema, and subarachnoid bleeding. The disease progressed rapidly, resulting in death within two years of diagnosis, highlighting the aggressive nature of this high-grade carcinoma with trophoblastic differentiation. Non-gestational trophoblastic tumors may represent a distinct disease group with unique clinical characteristics, and genetic analysis could help identify more cases.

## Introduction

There are three independent pathogenetic origins of trophoblastic differentiation in malignant tumors. One of these is the gestational trophoblastic neoplasms, which refers to a group of tumors that occur during pregnancy. Gestational trophoblastic neoplasms include invasive mole, which can be either a complete hydatidiform mole or partial hydatidiform mole, choriocarcinoma, placental-site trophoblastic tumor, and epithelioid trophoblastic tumor (1, 2). These tumors are characterized by containing paternal genetic material because of their relation to pregnancy (3).

The second are germ cell tumors of the ovary, testis, or other organs that can fully or partially contain trophoblastic elements (e.g., pure or mixed choriocarcinomas). These malignancies do not contain paternal DNA and are not associated with a prior gestation.

At last, partial trophoblastic differentiation has been found previously in some large-cell somatic carcinomas, e.g., endometrial or ovarian carcinomas, urothelial carcinomas, and lung carcinomas (4, 5). It is believed to develop through a clonal progression from the somatic components (3).

Non-gestational trophoblastic tumors are rare malignancies that arise independently of pregnancy and present diagnostic challenges. Patients may exhibit abnormal bleeding, pelvic masses, or metastatic symptoms. Diagnosis involves imaging techniques such as MRI and PET-CT, histopathology with markers like human chorionic gonadotropin (hCG) and placental alkaline phosphatase (PLAP), and molecular genetic testing, particularly short tandem repeat (STR) analysis, to differentiate between the two tumor types. Accurate differential diagnosis is crucial, as non-gestational trophoblastic tumors mimic other conditions such as gestational trophoblastic tumors, germ cell tumors, and high-grade carcinomas (6).

Recent advances have significantly improved non-gestational trophoblastic tumor management. STR analysis now provides precise differentiation, while immunohistochemical markers such as Ki-67 enhance tumor grading (7).

In this article, we share a case where a 38-year-old woman suffered from recurrent chest pain and shortness of breath and was diagnosed with a somatic non-gestational trophoblastic tumor.

## Case description

The study adheres to the CARE checklist criteria for quality reporting in case series (8).

Mrs. M.J. (38 years old, ECOG: 0) had no prior medical complaints. Regarding her gynecological history, she was a mother of two children (cesarean section delivery in 2017 and 2019). At the age of 22, a loop conization was performed (Papanicolau III). In April 2022, she began experiencing recurrent chest pain on the left side and periodic shortness of breath. Behind these symptoms, she had a case of drainage refractor pneumothorax, which has been followed with chest X-rays at a local healthcare facility. In April 2022, still at the local hospital, the patient underwent VATS biportal surgery for primary spontaneous pneumothorax, performing wedge resections from the left S1, S4, and S6 lung segments (9). Pathologically, there were no signs of malignancy in these samples. However, she experienced recurrent episodes of the symptoms, so in June 2022, a chest CT was performed, revealing a solid lesion in the lower left lobe and bullous degeneration in both lungs. Following this, the patient was referred to our institute for further evaluation and treatment.

**Figure 1:**
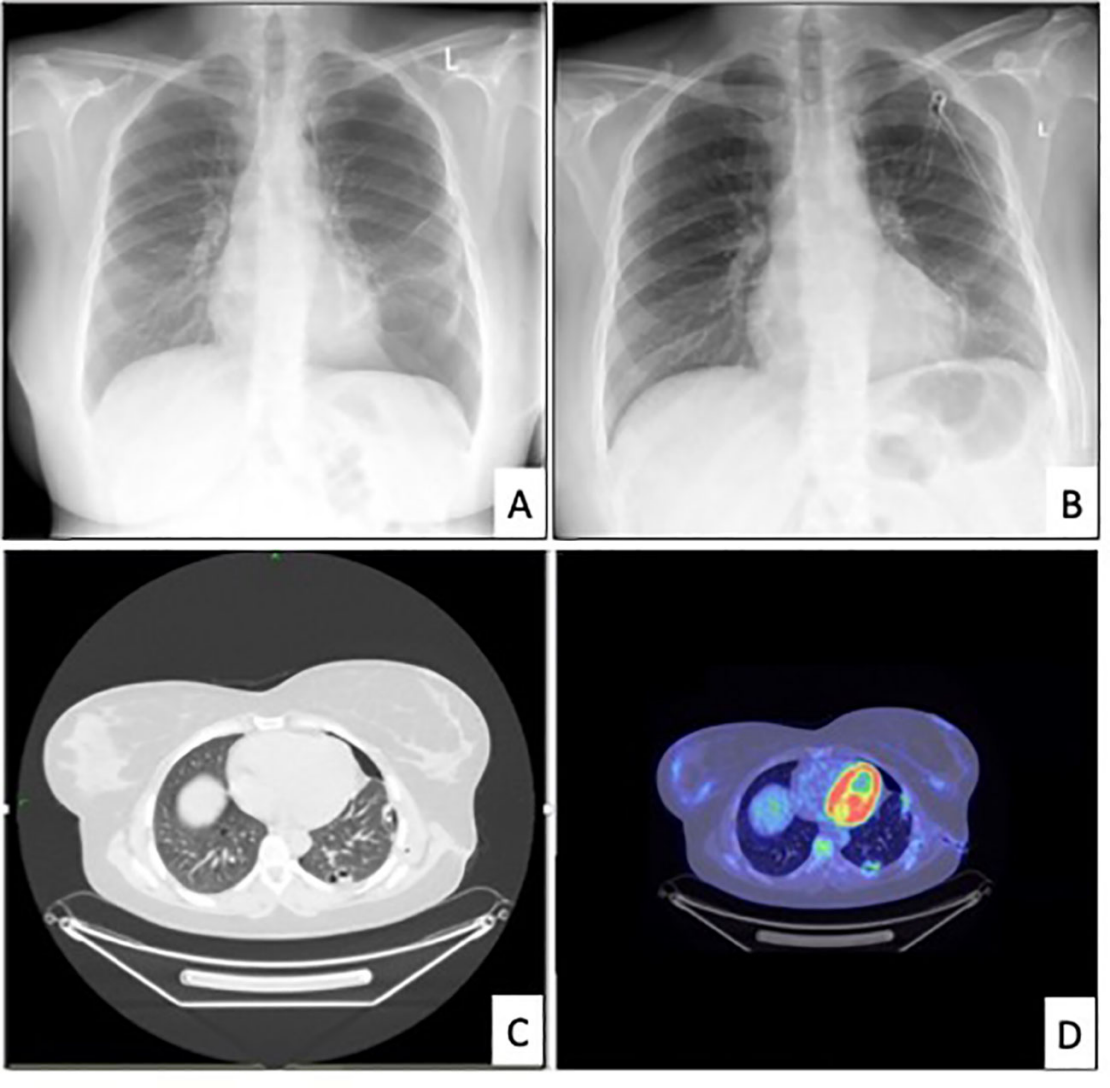
Relevant radiological images. [**(A)** chest x-ray revealing the recurrence of pneumothorax in June 2022. **(B)** chest x-ray after drainage, **(C)** chest CT in June 2022, **(D)** PET-CT in July 2022].

The patient underwent a standard radiological workup and oncological staging due to possible malignancy, with a subsequent PET-CT performed. In the left lung, a 2.2-1.4 cm lesion was found, surrounded by multiple subpleural bullae at the margin of segments 9-10. The scan indicated a slight accumulation of FDG with an SUV-max of 4.2. (**Figure 1**) An additional hilar lymph node was found with an SUV-max of 4.9, measuring 1 cm in size. There was residual postoperative activity with an SUV-max of 3.3 in the atelectatic region in the left lower lobe around the thoracic drain. FDG avidity in the right ovary (SUV-max of 10.8) was attributed to the menstrual cycle. (The later regular gynecological follow-up showed no progression in size or other characteristics regarding the ovaries.) Otherwise, the abdominal cavity and other parts of the body were negative on the PET scan.

**Figure 2:**
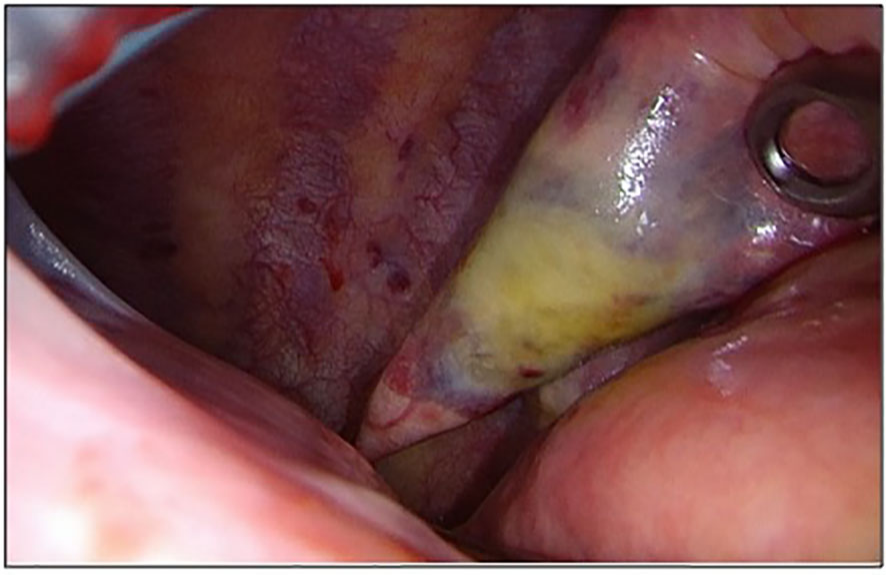
Intraoperative picture of the lesion in the left lower lobe.

In July 2022, the patient underwent a second VATS uniportal surgery to receive a diagnosis because of the FDG-avid lung lesion and the hilar lymph node. During the surgery, we discovered intermediate pleural adhesions and multiple diffuse bullous lesions in both of the lobes in the patient’s left lung. A solid component was also identified in the left lower lobe. (**Figure 2**) Apart from these findings, two additional pathological pleural lesions were detected. We removed the identified lesion in the lower lobe using a wedge resection, and intraoperative histology indicated a poorly differentiated carcinoma. A more specific histological diagnosis was not possible at this time. Due to the presence of multiple lesions and the nonspecific histological results, we did not perform an extended anatomical resection. However, we took additional biopsies from the upper lobe and pleural sites. We removed the drain on the first postoperative day and discharged the patient without any complications except for mild postoperative pain.

**Figure 3:**
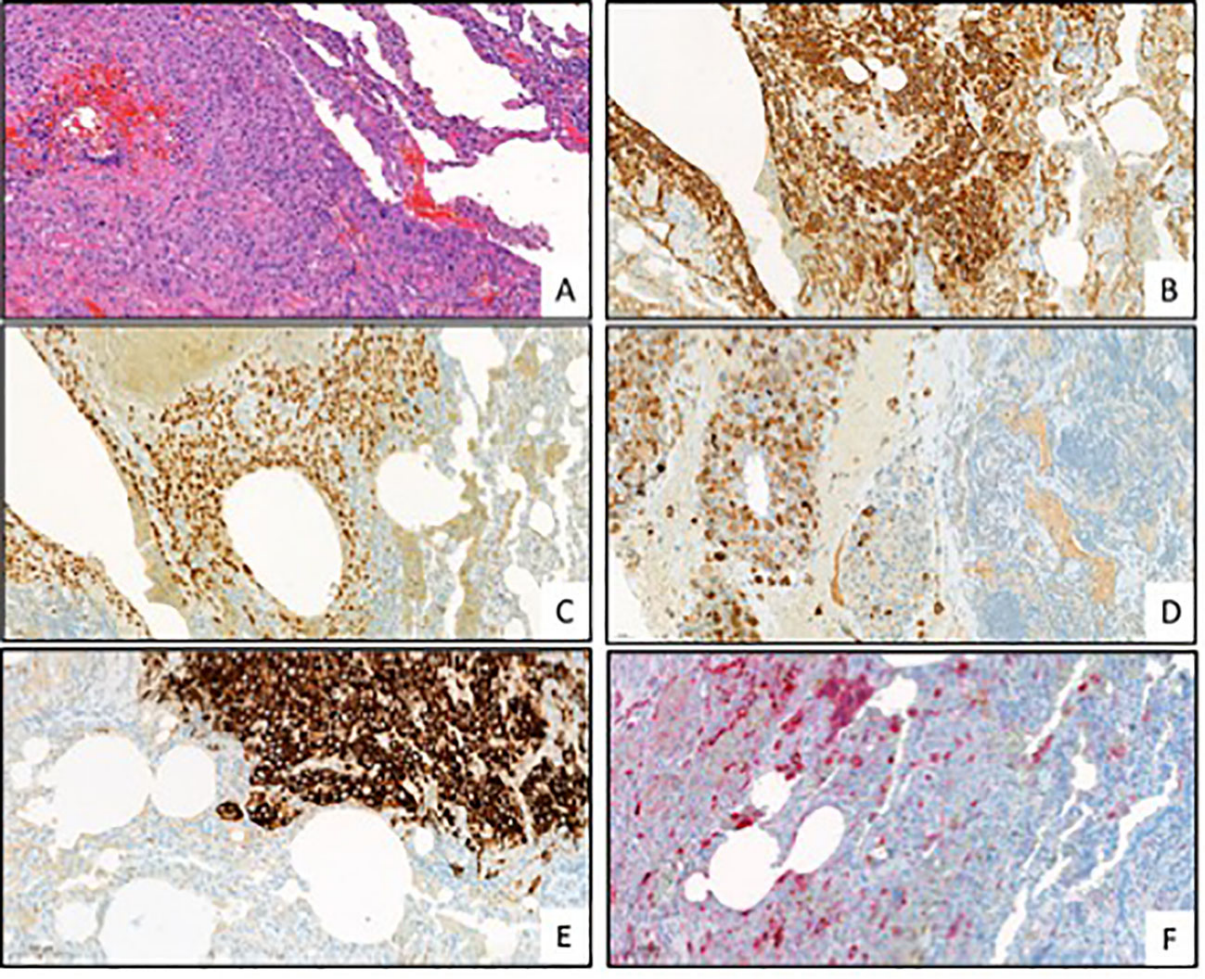
Representative pictures of the pathological evaluation. [**(A)** HE, **(B)** cytokeratin (CK), **(C)** GATA3, **(D)** human placental lactogen (hPL), **(E)** Mucin-4 (MUC4), **(F)** Human chorionic gonadotropin (hCG); all pictures are in 200x magnitude].

Following the surgical procedure, several medical evaluations were conducted to determine the origin of the primary tumor. The evaluations comprised a head/neck/abdominal MRI and consultations with dermatologists, otolaryngologists, and gynecologists. However, despite these efforts, none of the evaluations was able to identify a possible origin of the tumor. Upon pathological evaluation of the resected lung and pleural lesions, it was found that the histological samples contained homogeneous mononuclear cells with irregular hyperchromatic nuclei and eosinophilic cytoplasm. The immunohistochemical phenotype showed that CK, CK7, CK19, GATA3, and MUC4 were positive. The hPL and hCG reactions were also positive, while the inhibin reaction was positive only in a few cells. The beta-catenin was positive focally in a membranous form. However, the ER, PR, AR, CK5, p40, p63, TTF1, NapsinA, HEPA, synaptophysin, CK20, ERG, calretinin, CD34, PAX8, CAMTA1, CD31, CDX2, CDH17, SALL4, and vimentin reactions were negative. The PD-L1 tumor propensity score (TPS) was 95%, and the combined positive score (CPS) was 100. In addition, the microsatellite stability (MSS) and tumor mutation burden (TMB) were both low. The next-generation sequencing (NGS) 500 gene panel did not indicate any potential targets. The Ki-67 proliferation rate was 40%. Based on these findings, it was determined that the tumor had intermediate trophoblastic differentiation. The patient had a beta-hCG level of less than 1000 mU/ml (428.7), indicating that it could be a gestational-type trophoblastic tumor, mostly a placental site trophoblastic tumor. Short tandem repeat examination showed no signs of paternal DNA (AmpFlSTR MiniFiler PCR Amplification Kit), and there were no signs of 12p chromosome amplification or 12p isochromosome (Oncomine Comprehensive Assay Plus Panel). Therefore, the final diagnosis became a non-gestational high-grade somatic carcinoma with trophoblastic differentiation (**Figure 3**).

Initially, the tumor was believed to be a poorly differentiated pleomorphic, triple-negative large-cell carcinoma with a potential breast origin. It tested positive for GATA3 and had a PD-L1 CPS of 100. As a result, we began treatment with twelve cycles of paclitaxel (100mg/m2 on the first, eighth, and fifteenth day of each 28-day cycle following the Keynote-355 study protocol) along with pembrolizumab (200mg every twenty-one days) from September 2022 to December 2022 (10). Following the completion of twelve treatment cycles, the treatment regimen was simplified to include pembrolizumab as the sole therapeutic agent. Pembrolizumab was continuously administered alongside other treatments due to the massive PD-L1 positivity until October 2023. In April 2023, during a follow-up PET scan and abdominal MRI, two hepatic lesions that had not been previously detected were identified. A fast-track ultrasound-guided biopsy was performed, and histological samples were collected and analyzed. The pathological findings indicated that the liver and lung tumors were identical. In May 2023, we detected one cerebral metastasis and treated it with stereotactic radiation (1x18Gy). Due to the confirmed liver metastasis and the recent diagnosis of a non-gestational high-grade somatic carcinoma with trophoblastic differentiation, a change in treatment strategy was necessary. The EMA-CO/EP regimen, which consists of etoposide, methotrexate, actinomycin D, cyclophosphamide, vincristine, and etoposide, cisplatin with the latter two pair of agents alternating by each cycle, was implemented. This treatment was continued until October of 2023, when it was discontinued due to severe myelotoxicity. In the same month, the patient developed a pulmonary empyema, and a CT scan revealed additional hepatic, renal, and pancreatic propagation. Considering the patient’s low HER2 status, we planned to initiate trastuzumab-deruxtecan therapy at a dose of 5.4 mg/kg/21 days. Unfortunately, multiple cerebral metastases occurred, accompanied by subarachnoid bleeding. Due to the complications arising from these conditions, the patient passed away in December 2023 (**Table 1**).

**Table 1:** Timeline of events.

Date	Event
**April 2022**	Initial symptoms of recurrent chest pain and shortness of breath.
	Diagnosed with pneumothorax and underwent multiple thoracic drainages.
	VATS biportal surgery for primary spontaneous pneumothorax with no malignancy in samples.
**June 2022**	Chest CT revealed a solid lesion in the lower left lobe and bullous degeneration in both lungs
**July 2022**	Referred to our institute for further evaluation.
	PET-CT scan: 2.2-1.4 cm lesion in left lung, FDG avidity, additional hilar lymph node with SUV-max of 4.9.
	VATS uniportal surgery: discovered pleural adhesions, bullous lesions, and solid component in the left lower lobe.
	Pathology: poorly differentiated carcinoma, additional biopsies taken. Homogeneous mononuclear cells, positive immunohistochemical markers (CK, CK7, CK19, GATA3, MUC4, etc.), high PD-L1 score, low MSS and TMB.
	Diagnosed with non-gestational high-grade somatic carcinoma with trophoblastic differentiation
**September 2022**	Initiated treatment with paclitaxel and pembrolizumab
**December 2022**	Completed 12 cycles of paclitaxel; continued pembrolizumab monotherapy.
**April 2023**	Follow-up PET scan and abdominal MRI revealed two hepatic lesions.
	Biopsy confirmed liver and lung tumors were identical
**May 2023**	Detected cerebral metastasis; treated with stereotactic radiation.
	Changed treatment strategy to EMA-CO/EP regimen due to confirmed liver metastasis and diagnosis of carcinoma with trophoblastic differentiation.
**October 2023**	Discontinued EMA-CO/EP regimen due to severe myelotoxicity.
	Developed pulmonary empyema and further hepatic, renal, and pancreatic metastases.
	Planned trastuzumab-dexrutecan therapy.
**December 2023**	Patient passed away due to complications from multiple cerebral metastases and subarachnoid bleeding.

The bold text indicates the month in which disease progression occurred and when interventions or therapeutic modifications took place.

## Discussion

Trophoblastic tumors are rare and, as mentioned above, often present a significant diagnostic challenge (11). Gestational trophoblastic tumors commonly lead to pulmonary metastasis (average 60-80% of all cases) and occasionally present primarily with pneumothorax. However, according to the recent FIGO/WHO or Dutch clinical classification system, gestational trophoblastic tumors with or without lung metastasis usually have a good prognosis and respond well to treatment, with a healing rate of almost 100% (12, 13) High-risk gestational trophoblastic tumor patients are commonly treated with multi-agent regimens, such as EMA-CO (etoposide, methotrexate, actinomycin D, cyclophosphamide, and vincristine). EMA-CO has shown complete response rates of 71-78% and long-term survival rates of 85-94% (14).

There have been a few cases reported in the medical literature of gestational trophoblastic disease with metastasis to the thorax outside the lungs, including some intracardiac metastasis, which are associated with varying prognoses (15). If a woman of reproductive age is diagnosed with an extra-uterine trophoblastic tumor, it will most likely be assumed to be gestational. There are three possible explanations for the tumor not being gestational in origin. First, it could have metastasized from an undetected or hidden uterine or ovarian tumor. Second, it could be related to an undetected ectopic gestational event if it is found in sites other than the ovary, fallopian tube, or the uterus (2). At last, a somatic tumor with trophoblastic differentiation. It is important to note that the first is an example of germ cell origin, and the second, although often referred to as non-gestational, does not exhibit the characteristic features of such tumors. It is unclear whether gestational and non-gestational trophoblastic tumors have different behaviors and treatment requirements due to their distinct genetic profiles (16). However, the current experiences suggest that somatic-type trophoblastic tumors react differently to treatment and result in poor prognosis, unlike the gestational types (3).

Somatic non-gestational trophoblastic tumors are sporadic, and the case where they manifest primarily in a distant region from the female genitals is even rarer. Based on the literature, previous cases occurred in the lungs or the liver (17, 18). However, we assume there needs to be more accuracy in the medical literature regarding the terminology for these certain types of tumors expressing trophoblastic markers. The terms “primary choriocarcinoma of the lungs,” “large cell lung carcinoma with a trophoblastic marker,” and “combined lung choriocarcinoma and adenocarcinoma” have overlapping definitions and can be confusing. For example, in 2006, Yamato et al. published 31 cases of lung choriocarcinoma, while during the same period, Chen et al. reported 12 additional cases of combined choriocarcinoma and adenocarcinoma (19). In 2020, Wu et al. stated that primary choriocarcinoma of the lung is extremely rare, with fewer than 30 cases reported to date (4, 20). The prognosis and response to treatment for these examples vary greatly, further supporting the idea that they are not homogenous.

It is essential to differentiate somatic forms of trophoblastic tumors. Only a few reported cases can be found in the literature when the tumor originates in the lungs. For example, Buza et al. reported similar cases involving three patients; two were gestational in origin, and one was somatic, primarily coming from the lungs. This last patient showed a poorer response to the treatment and a less favorable prognosis (3). Somatic-type trophoblastic tumors seem to react to treatment differently in general, even in other organs (21). To the best of our knowledge, we believe that we published the first case where the leading symptom of a non-gestational trophoblastic tumor with somatic origin was recurrent pneumothoraces. Due to the lack of homogeneity, we believe primary somatic trophoblastic lung tumors could be more common, and their general incidence could be much higher. However, it is yet unknown (16).

## Conclusion

A review of the literature reveals case reports about choriocarcinoma of the lung, poorly differentiated trophoblastic tumors of other organs, and other trophoblastic marker-expressing tumors that may represent similar entities. However, we may fail to recognize these similarities due to the lack of homogeneous genetic testing. Diagnosing non-gestational trophoblastic tumors and distinguishing them from their gestational counterparts is challenging, as the recent WHO classification does not assign them to a particular group. Incorporating non-gestational tumors based on their genetic characteristics into the female genital tumor classification system may aid in their diagnosis (22).

This report presents a rare case of a non-gestational trophoblastic tumor primarily detected in the lung. Our findings have implications for highlighting the possible underdiagnosis of these cases and show the importance of proper diagnosis and management of these tumors. We hope this report provides valuable information for clinicians and researchers, contributing to the growing knowledge of trophoblastic tumors.

## Data Availability

The original contributions presented in the study are included in the article/**Supplementary Material**. Further inquiries can be directed to the corresponding author.
